# Some Isolated Cardiac Malformations Can Be Related to Laterality Defects

**DOI:** 10.3390/jcdd5020024

**Published:** 2018-05-02

**Authors:** Paolo Versacci, Flaminia Pugnaloni, Maria Cristina Digilio, Carolina Putotto, Marta Unolt, Giulio Calcagni, Anwar Baban, Bruno Marino

**Affiliations:** 1 Department of Pediatrics, Sapienza University of Rome, 00161 Rome, Italy; paolo.versacci@uniroma1.it (P.V.); flaminia.pugnaloni@gmail.com (F.P.); caro_85@hotmail.it (C.P.); unolt.marta@gmail.com (M.U.); 2 Genetics and Rare Diseases Research Division, Bambino Gesù Children’s Hospital and Research Institute, 00165 Rome, Italy; mcristina.digilio@opbg.net; 3 Department of Pediatric Cardiology and Cardiac Surgery, Bambino Gesù Children’s Hospital and Research Institute, 00165 Rome, Italy; giulio.calcagni@opbg.net (G.C.); anwar.baban@opbg.net (A.B.)

**Keywords:** congenital heart disease, genetics, heterotaxy, atrioventricular canal defect, transposition of the great arteries

## Abstract

Human beings are characterized by a left–right asymmetric arrangement of their internal organs, and the heart is the first organ to break symmetry in the developing embryo. Aberrations in normal left–right axis determination during embryogenesis lead to a wide spectrum of abnormal internal laterality phenotypes, including *situs inversus* and *heterotaxy*. In more than 90% of instances, the latter condition is accompanied by complex and severe cardiovascular malformations. Atrioventricular canal defect and transposition of the great arteries—which are particularly frequent in the setting of *heterotaxy*—are commonly found in *situs solitus* with or without genetic syndromes. Here, we review current data on morphogenesis of the heart in human beings and animal models, familial recurrence, and upstream genetic pathways of left–right determination in order to highlight how some isolated congenital heart diseases, very common in *heterotaxy*, even in the setting of *situs solitus*, may actually be considered in the pathogenetic field of laterality defects.

## 1. Introduction

Human beings, like other vertebrates, are characterized by a left–right asymmetric arrangement of their internal organs. The initial left–right asymmetry is generated by the rotational movement of cilia at the primitive node post-gastrulation of the mammalian embryo and have a whirling, unidirectional clockwise rotation, generating an asymmetric leftward flow of extraembryonic fluid and breaking the bilateral symmetry of the embryo [1,2]. The nodal cilia play an essential role in the establishment of left–right patterning during embryonic development, and their aberrations can bring laterality defects [3,4]. Abnormalities in normal left–right axis determination during embryogenesis lead to a wide spectrum of abnormal internal laterality phenotypes, including *situs inversus* and *heterotaxy,* also termed *situs ambiguus*. While *situs inversus* is characterized by a complete mirror-imaged arrangement of the internal organs along the left–right axis, *heterotaxy* is defined as any arrangement of internal thoracic and abdominal organs other than *situs solitus* or *situs inversus*. It is characterized by a wide variety of cardiac and extracardiac congenital malformations, which are primarily induced by disorders of the left–right axis determination during early embryonic development. In patients with *heterotaxy*, the genetic message of visceral asymmetry is partially or completely lost with the lack of definitive positional information, and morphologic symmetry of some organs is characteristic, such as atrial appendages, bronchi, and lungs. The latter aspect gave rise to a definition of these conditions as *atrial isomerism* or, more correctly, *isomerism of the atrial appendages*, identifying two main subtypes: *right isomerism*, with both trilobed lungs with short eparterial bronchi, both atria with right atrial appendages and absence of the spleen (*asplenia*); and *left isomerism*, with both bilobed lungs with long hyparterial bronchi, both atria with left atrial appendages, and two or more splenic masses in the abdomen (*polysplenia*). *Heterotaxy* is accompanied in more than 90% of instances by complex and severe cardiovascular malformations and frequently by cardiac malposition, such as *mesocardia* or *dextrocardia*, supporting the fact that the heart appears particularly sensitive to perturbation in normal left–right positional information. Patients with *asplenia/right isomerism* phenotype typically have more severe and various combinations of cardiac defects. On the contrary, *polysplenia/left isomerism* phenotype is usually characterized by less severe cardiac malformations (Table 1).

Abnormalities in abdominal organ distribution can have specific clinical and prognostic implications in terms of predisposition to intestinal malrotation and immune deficiency in some asplenic patients [5,6].

In the last two decades, our group has been deeply involved in the research of congenital heart diseases (CHDs) related to aberration of left–right patterning during cardiac morphogenesis. Some congenital cardiovascular malformations that are particularly frequent in the setting of *heterotaxy*, such as atrioventricular canal defect (AVCD) and transposition of the great arteries (TGA), are prevalently found in *situs solitus* with or without genetic syndromes. In 1998, Brett Casey, one of the pioneers in the field of genetics of *heterotaxy*, asked: “Are some complex, isolated heart malformations actually unrecognized manifestations of aberrant left–right asymmetry development?” [7]. This question was based on clinical and genetic observations, which suggested not only that familial *heterotaxy* occurred with autosomal dominant (usually with incomplete penetrance), recessive, and X-linked inheritance [8,9], but also that some isolated CHDs appearing in relatives of individuals with *heterotaxy* would be linked to laterality defects. Due to the continuous increase of novel approaches (Next-Generation Sequencing—NGS, exome sequencing, genome sequencing), our understanding of the molecular basis of AVCD and TGA is progressively growing. Table 2 shows a summary of human genes associated with AVSD and TGA, and points out the possible mechanism underlying the observed phenotype.

The aim of this review, based upon morphology, familial recurrence, experiments on animal models, and genetic data, is to highlight how some isolated *heterotaxy*-like CHDs, even in the setting of *situs solitus,* can be related to laterality defects.

## 2. Atrioventricular Canal Defect

**Figure 1:**
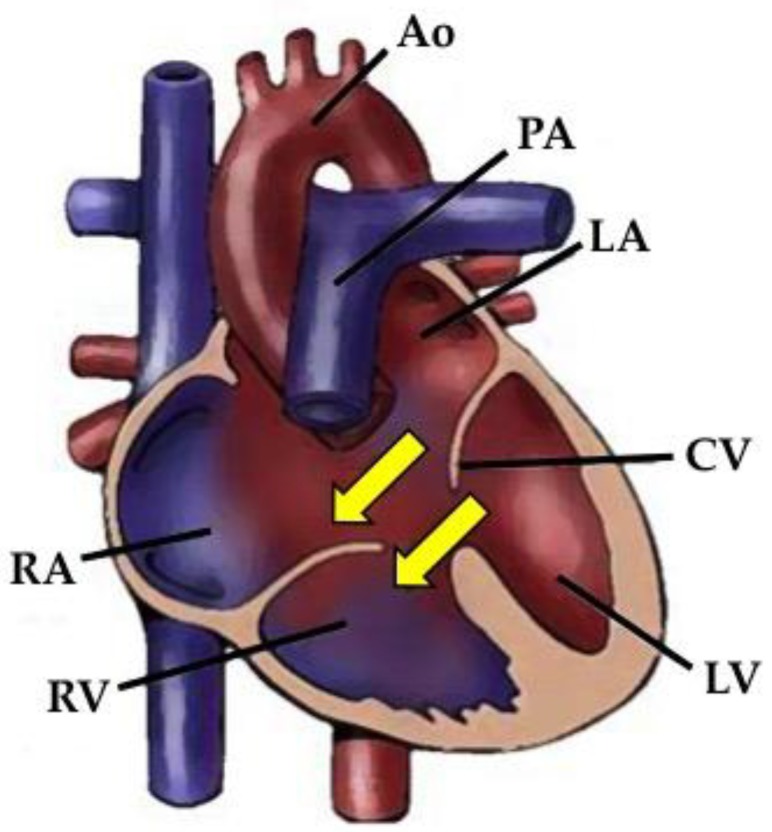
The diagram shows the complete form of AVCD with an ostium primum atrial septal defect, a ventricular septal defect, and a single common atrioventricular valve. The yellow arrows represent the left-to-right shunt through atrial and ventricular septal defects. RA: right atrium; RV: right ventricle; LA: left atrium; CV: common valve; LV: left ventricle; Ao: aorta; PA: pulmonary artery.

Atrioventricular canal defect (AVCD) is also termed atrioventricular septal defect or endocardial cushion defect, and covers a spectrum of CHDs that result from failure of the ventral (antero-superior) and dorsal (postero-inferior) endocardial cushions to fuse properly. It has an incidence of 3.5 per 10,000 live births and represents about 7.4% of all CHDs [10]. The anomalies that characterize AVCD involve atrioventricular valves and atrial and ventricular septa. AVCD can be classified as complete, partial, or intermediate. The complete form of AVCD is characterized by a single common atrioventricular valve, an ostium primum atrial septal defect, and a confluent posterior ventricular septal defect in the inlet portion of the ventricular septum (Figure 1). The partial form of AVCD includes two separate (right and left) atrioventricular valves with a *cleft* of the antero-medial leaflet of the mitral valve, an ostium primum atrial septal defect, and no ventricular septal defect. The intermediate form is like the partial AVCD, but with a restrictive posterior ventricular septal defect [11].

AVCD may occur in otherwise normally-developed infants in the so-called isolated or non-syndromic form, representing 25% of affected individuals [12]. However, AVCD is often associated with congenital extracardiac malformations and genetic syndromes [10,12]. In fact, in the Caucasian population, it is the “classic” CHD in Down syndrome accounting for 45% of total cases [13,14,15]. The second specific association is with *heterotaxy* (15% of cases), and other genetic syndromes account for an additional 15% of cases [12].

### 2.1. AVCD Embryology

Despite a large number of investigations, the morphogenesis and the genetic basis of AVCD are still not fully understood. Traditionally, it was thought that the only mechanism leading to AVCD was the perturbation of the fusion of the endocardial cushions in relation to defects of extracellular matrix [16,17]. However, recent observations of the human heart and an increasing number of experimental studies have revealed that abnormal development of tissue of extracardiac origin derived from the posterior second heart field (SHF) and called the dorsal mesenchymal protrusion (DMP) plays a pivotal role in the pathogenesis of AVCD [11,18,19,20,21,22,23]. The origin of this structure is a matter of some controversy, but its importance in atrial and atrioventricular septation now appears to be well established. The DMP, formerly named “spina vestibuli” [24,25,26], was initially described by His in 1880 as a triangular mesenchymal wedge which protruded into the lumen of the atrium from a nonmuscular area, which he called the “area interposita” in the dorsal wall of the common atrium [27,28,29]. At present, we know that it originates in the splanchnic mesoderm ventral to the foregut as a mesenchymal protrusion that contributes to the final formation of the basal part of the developing atrial septum [30]. In direct continuity with the mediastinal mesenchyme, this structure protrudes into the atrial cavity at the caudal end of commissure between the valves of the *sinus venosus*. Normal growth of the DMP is important for separate formation of the right and left atrioventricular junction [31,32]. When development proceeds normally, the DMP grows to reinforce the right side of the area over which an endocardial cushion-like structure lining the free under-rim of the septum primum, the so-called mesenchymal cap, fuses with the atrial surface of atrioventricular endocardial cushions [28]. The fusion of the mesenchymal tissues with the atrioventricular endocardial cushions leads to the closure of the ostium primum [33]. Failure of the endocardial cushions, mesenchymal cap, and DMP to fuse to one another properly results in a common atrioventricular valve and an ostium primum atrial septal defect.

In this context, it is important to highlight the pivotal role of the homeobox transcription factor *Pitx2* in the morphological left atrial identity, confirmed by the observation that *Pitx2* knock-out mice display a complex cardiac phenotype which is typical of *right isomerism* [34]. To date, no gene has been able to drive the right atrial identity, which is considered as a “default” state of the sinoatrial region cardiomyocytes that can be turned into left only by the local action of *Pitx2* [35]. Both myocardial cells of the interatrial septum and cells in the left (but not right) atrial wall express *Pitx2*, indicating a molecular asymmetry which is present in the common atrium prior to its differentiation [36,37,38]. It is intriguing to mention that fish hearts are characterized by a common atrium, which does not present left–right morphological differences, suggesting that the acquisition of a morphological left–right identity is correlated with the presence of pulmonary circulation [35]. Moreover, myocardium in the atrioventricular canal also exhibits left- but not right-side *Pitx2* expression [39,40,41]. These sorts of cellular and molecular left–right differences suggest that, similar to the common atrium, the atrioventricular canal region is also lateralized [38].

### 2.2. Cilia and Sonic Hedgehog Signaling Pathway

Cilia are evolutionarily conserved organelles and, on the basis of the arrangement of the microtubules of the central axoneme, have been historically divided into two main forms: motile and primary or sensory. Presently, there are three broad classes of cilia: motile 9 + 2, which consists of nine microtubule pairs arranged in a circle and surrounding a central pair; motile 9 + 0, which lack the central pair of microtubules; non-motile or primary 9 + 0 [42]. While the 9 + 2 motile cilia beat in a synchronous waveform, the 9 + 0 motile cilia are present only transiently at the primitive node post-gastrulation of the mammalian embryo.

Cilia protrude from the apical surface of most vertebrate cell types and serve a multitude of functions, including signaling, extracellular fluid propulsion, and sexual reproduction. Motile cilia are found on the apical surface of the upper and lower respiratory tract, on the ependymal cells that line the ventricles of the brain, and in the oviducts [43]. Defects affecting the structure and/or function of cilia can lead to several human genetic diseases with overlapping phenotypes—the so-called “ciliopathies”. Kartagener’s syndrome, which is part of a group of diseases called primary ciliary dyskinesia (PCD), is a typical example of a human condition associated with abnormal function of motile cilia, and is characterized by bronchiectasis, infertility, and in about 50% of cases by *situs inversus*. Mutations of *DNA11* and *DNAH5*, which encode for dynein arms that link the microtubules of normal motile cilia, are a cause of *heterotaxy* in 6.5% of patients with PCD [44]. Similarly, NGS identified mutations in *DNA11*, *DNAH5*, and *DNAH11* in 13 patients with ciliary dysfunction and *heterotaxy* [45]. Vetrini et al. [46] identified homozygous mutations in *PKD1L1* (polycystic kidney disease like 1 gene) from three affected individuals in two unrelated families (one with *situs solitus*, one with *heterotaxy*, one with *situs inversus*), and all of them had complex CHDs. The gene encodes a polycystin-1-like protein, which is involved in fluid-flow mechanosensation by the primary cilium in renal epithelium, and its loss of function is known to cause laterality defects in mouse models [47,48].

Cilia are structurally present in the SHF, and recent experimental data on animal models have demonstrated that atrioventricular septation and DMP development require cilia-based Sonic hedgehog (*Shh*) signaling, and the primary cilium is required for Hh signaling [49]. *Shh* signaling is a fundamental cilia-transduced cell signaling [50], and it is used constantly for intercellular communication during the development of almost every organ in vertebrates [51]. Vertebrate *Shh* signaling is completely dependent on the primary cilium, and the experimental ablation of cilia can result in a drastic reduction of *Shh* signaling [50]. *Shh* is the ligand, which acts through the membrane receptor Patched (PTCH1) localized on the distal end of the axoneme. When the ligand is absent, the *Shh* receptor PTCH1 keeps the pathway off, inhibiting the activity of the seven transmembrane-domain protein Smoothened (SMO). When SMO is inactive, three different transcription factors, glioma-associated oncogenes 1 to 3 (*Gli1*-*3*), are proteolytically processed to make a transcriptional repressor that binds to *Shh* target genes and blocks their transcription. Binding of *Shh* to PTCH1 inhibits its activity, reducing the repression of SMO, which promotes the conversion of full-length GLI into a transcriptional activator [51]. The *Shh* cascade is crucial for regulating SHF contribution to the cardiac inflow and outflow tracts and to cardiac septation [21,52,53]. Goddeeris et al. [53] demonstrated that *Shh* signaling is required within the dorsal mesocardium for its contribution to atrial septation, and a failure of this addition results in a severe form of AVCD. In the study of Burnicka-Turek et al. [49], the central role for cilia in AVCD is elucidated, clarifying the relationship between primary cilia genes, *Shh* signaling, and AVCD. Intraflagellar transport (IFT) is a system composed of 20 proteins that is required for building primary cilia [54]. Mouse lines carrying mutations in IFT genes display several CHDs, including AVCD. The *Ift25* mutant embryos studied in the study of Keady et al. [55] showed that *Ift25* is not required for ciliary assembly, but defective mutants display phenotypes of typical *Shh* signaling defects, including polydactyly, cleft palate, lung isomerism, and AVCD. Due to in vivo analysis, it was postulated that *Ift25* is necessary for the dynamic transport of target *Shh* genes (*Gli2*) at the ciliary tip in response to activation of the *Shh* pathway. Thus, IFT function plays a role in cilium signal transduction events. Another component of the IFT system is *Ift88*, which is essential for the formation of primary cilia. In fact, mutant mice that carry a hypomorphic allele for *Ift88* show reduced numbers of cilia and weakened *Shh* signaling, indicated by decreased up-regulation of *Shh* targets such as *Gli1* and PTCH1. This data further points to an interesting link of AVCD to genes of the primary cilia [56,57]. Among IFT genes, *Ift172* is also included, which encodes a subunit of the intraflagellar transport subcomplex IFT-B. In mice models, null allele of *Ift172* results in absent cilia and loss of *Shh* signaling because IFT proteins are required for both *Gli* activator and *Gli* repressor function [58,59]. Mutations in *Ift140* have been identified in skeletal human ciliopathies such as Jeune syndrome. Miller et al. [60] found that embryos harboring a homozygous *Ift*140 null allele exhibit phenotypes including abnormal atrioventricular valves and defective interventricular septum. In the primary cilium, a key role is carried out by *Fuz*, which encodes a planar cell polarity protein (pcp) involved in ciliogenesis. *Fuz* knockout mice display a large cohort of cardiac defects including AVCD [61,62], in addition to neural tube defects, skeletal malformations, and defective cilia. *Fuz* is essential to ciliar membrane trafficking, so mutant mice show disrupted ciliogenesis and sequential *Shh* signaling defects [59]. Another important gene demonstrated to be causative of AVCD in mice models is *Kif7*, which belongs to the kinesin family and encodes a cilia-associated protein. This protein regulates the *Shh* signaling pathway, acting downstream of SMO and upstream of *Gli2*. *Kif7* generally localizes to the base of the primary cilium and functions as a negative regulator of the *Shh* pathway in the absence of the correct ligand, but also as a positive regulator of the same pathway blocking the repressor form of *Gli3* [63,64,65]. Mutations that disrupt the structure of the primary cilium centrosome also create a dysregulation of the *Shh* pathway. Indeed, Chen et al. [66] demonstrated that mutants of Cp110(Cntrl), a centrosomal protein, show aberrant formation of primary cilium [67]. Interestingly, after *N*-ethyl-*N*-nitrosourea (ENU) mutagenesis, the mutant mouse Cntrl^b2b1468.1Clo^/Cntrl^b2b1468.1Clo^ was found to have a variety of cardiac defects, including AVCD [68]. However, how Cp110 inhibits the ciliogenesis program is still unknown.

In the primary cilium trafficking of *Shh*, components pass through a sort of “ciliary gate”, mostly composed by the transition zone [69]. A protein encoded by the gene *Mks* is located in this important region. This protein is normally required for the formation of the primary cilium in epithelial cells. Mutations in this gene result in Meckel syndrome type 1, which is characterized by severe defects of the cerebellar vermis and CHDs including AVCD [70]. In the study of Aguilar et al. it is demonstrated that the cerebellar phenotype is dependent on the dramatic reduction of the proliferation of granule cell progenitors due to disruption of the *Shh* pathway. However, further studies on its effect on cardiogenesis are needed [70].

### 2.3. Syndromic AVCD Related to Sonic Hedgehog Pathway

Mutated genes that are responsible for several syndromes with AVCD are causally involved in ciliary dysfunction and/or abnormal processing of proteins participating in *Shh* signaling. Ellis–van Creveld syndrome is an autosomal recessive disorder characterized by short-limb dwarfism, short ribs, postaxial polydactyly of hands and feet, and ectodermal defects, and is due to mutations in *EVC* and *EVC2* genes, which are intracellular components of the *Shh* signal transduction pathway. AVCD associated with common atrium and bilateral superior vena cava with unroofed coronary sinus are the most common CHDs in this syndrome, recalling the atrial morphology of patients affected by *heterotaxy*. Interestingly, AVCD with common atrium is characteristic of other ciliopathies associated with postaxial polydactyly, including Bardet-Biedl syndrome, oral–facial–digital syndrome, and short rib polydactyly syndromes [71]. These syndromes occur due to mutations in genes whose proteins are involved in ciliary function regulation [72,73,74,75]. AVCD often associated with anomalous pulmonary venous drainage is typical of Smith–Lemli–Opitz syndrome (SLOS), which is a congenital multiple anomaly syndrome, inherited in an autosomal recessive manner. Clinical manifestations of SLOS include mental retardation, microcephaly, growth retardation with feeding difficulties, facial anomalies, cataract, cleft palate, hypospadias, 2–3 toe syndactyly, and postaxial polydactyly. It is caused by an inborn error of cholesterol biosynthesis leading to low plasma cholesterol levels and elevated concentrations of the cholesterol precursor 7-dehydrocholesterol, due to a deficiency of the enzyme 7-dehydrocholesterol reductase (DHCR7) [76,77]. Cholesterol has a critical role in the formation of the normally active *Shh* proteins [78]. Abnormal processing of *Shh* proteins secondary to abnormal cholesterol levels seems to have a role in the development of SLOS malformations [78,79]. In VACTERL (vertebral defects, anal atresia, cardiac defects, tracheo–esophageal fistula, renal anomalies, and limb abnormalities) association, CHDs occur in 50–80% of the cases, mainly septal defects. However, CHDs in VACTERL do include cardiac laterality defects such as *dextrocardia*, *heterotaxy*, AVCD, and TGA [12,80,81]. The etiology is considered to be multifactorial with environmental influences [81]. Mice with mutations in *Shh* pathway genes (e.g., *Shh* and *Gli* genes) display a spectrum of defects resembling the human VACTERL association, suggesting that some or most of the VACTERL phenotypes in human beings could be explained by aberrations in *Shh* signaling [82,83]. A mutation in the *FOXF1* gene, which is linked to *Shh* signaling, results in a VACTERL-like phenotype, and mutation in the *HOXD13* gene, which is a downstream target of *Shh*, has been described in a patient with features of VACTERL association [84,85,86]. Moreover, a study of the *atrioventricular canal 1* (*avc1*) mouse mutant—a mouse mutation that caused VACTERL association with hydrocephalus (VACTERL-H)—showed AVCD in 100% of *avc1* mutants analyzed. *avc1* is a hypomorphic mutation of intraflagellar transport protein 172 (*Ift172*), which is required for ciliogenesis and *Shh* signaling [58].

Recent observations in mouse models have pointed out the role of the *Shh* signaling pathway in Down syndrome. These experiments have demonstrated a defective mitogenic *Shh* activity in trisomic cells of brain, skin, liver, and intestine of mice, with cell proliferation impairment due to a higher expression of PTCH1, which is the receptor that normally represses *Shh* signaling [87]. This suggests that PTCH1-dependent inhibition of *Shh* signaling may underlie proliferation impairment in trisomic peripheral tissues, leading to defective neuronal production in the brain of Down syndrome patients [88]. Moreover, subcutaneous administration of a *Shh* pathway agonist known as SAG to trisomic Ts65Dn mice at birth resulted in an increased proliferation of granule cell precursors in the cerebellum [89]. In another recent study, Ripoll et al. [90] suggest that AVCD and other CHDs found in Down syndrome patients may be associated with an altered ciliome. In conclusion, the majority of genetic syndromes associated with AVCD are due to genetic mechanisms in relation to lateralization defects involving *Shh* pathway and ciliopathy.

### 2.4. Isolated or Non-Syndromic AVCD

Accordingly to the multifactorial model of inheritance [91], in families of patients with isolated or non-syndromic AVCD, the frequency of recurrent CHDs in siblings is relatively higher compared with patients with other types of CHDs, reaching 3–4% [91,92,93,94,95]. In agreement with the literature and in our experience, recurrent CHD in the family was prevalently concordant [92]. In fact, many pedigrees showed vertical transmission of AVCD, including complete and partial forms and isolated cleft of the mitral valve, suggesting pathogenetic similarities between these CHDs [92,96]. This pattern of recurrence indicates an autosomal dominant mechanism with monogenic or oligogenic inheritance in selected pedigrees, suggesting biochemical pathways of interest [92,96,97,98,99,100,101,102]. However, AVCD is a genetically heterogeneous heart malformation, and in human beings it very infrequently occurs as a single-gene defect. It is noteworthy that affected mothers seem to have a higher risk of transmitting AVCD in comparison with affected fathers, suggesting a mitochondrial inheritance among etiological mechanism. In fact, the recurrence risk rate for offspring becomes 14% when the affected parent is the mother [103,104]. In terms of well-known environmental factors, strong associations between complete AVCD and maternal diabetes [105], pregestational diabetes, gestational diabetes and obesity (BMI >30 kg/m^2^) [106], and between AVCD and heavy smoking have been described [107]. All these observations suggest that epigenetic factors could contribute to the pathogenesis of AVCD [108].

Several in vivo and in vitro studies have identified multiple genetic mutations that have the potential to cause AVCD. Different genes underlying isolated AVCD disrupt *Shh* signaling, but the central role of primary cilia in cardiogenesis is also emerging in regard of this malformation.

One of the first candidate genes to be identified was *CRELD1*, an important regulator of the calcineurin/NFATC1 signaling, mapping to chromosome 3p25, in the AVCD2 locus. Although missense mutations of *CRELD1* were found in Down syndrome and *heterotaxy*, further studies demonstrated that this gene also plays an important role in the pathogenesis of isolated AVCD, occurring in about 5% of non-syndromic cases [92]. Moreover, family studies on missense mutation of *CRELD1* are consistent with incomplete penetrance of this CHD. Inactivating mutations of *CRELD1* interfere with VEGF-dependent epithelial-to-mesenchymal transformation—a crucial step in the morphogenesis of the atrioventricular endocardial cushions into the mature valve [109,110]. Interestingly, *CRELD*^+/−^ mice do not have septal defects, but when they are crossed onto “Down syndrome mice” Ts65Dn, they develop septal defects [23]. In a large number of unrelated affected individuals with AVCD, a causal mutation in *NR2F2* has been identified, which encodes a member of the steroid/thyroid hormone superfamily of nuclear receptors [111]. This gene is involved in organogenesis of the stomach, limbs, skeletal muscles, and heart. *NR2F2* mutations have been associated with isolated and familial AVCD cases, supporting the hypothesis that this gene is implicated in endocardial cushion development and specifically that cardiac development is likely to be sensitive to the dosage of functional *NR2F2* [112,113]. In the study of Li et al., a possible link between *NR2F2* and *Shh* signaling has been proposed, since it was demonstrated that *Shh* could regulate the angiogenic growth factor pathway by mediating the nuclear receptor *NR2F2* [112]. *GATA4* stands out among genes involved in embryogenesis and in myocardial differentiation. It encodes a member of the GATA family of zinc-finger transcription factors. In humans, non-synonymous *GATA4* variants were associated with AVCD, in addition to a wide variety of other CHDs such as atrial septal defects and ventricular septal defects. The study of Zou et al. [114] gave evidence of the unexpected relationship between *GATA4* and *Shh* signaling, demonstrating that *GATA4* deletion in the SHF results in a failure of DMP formation and cell-cycle progression of cardiac progenitors in the posterior SHF.

Some genes deeply implicated in syndromic CHD can contribute to the etiology of isolated cardiac malformations. Among these genes there is *PTPN11*, whose mutations are causative of almost 50% of Noonan syndrome not rarely presenting with AVCD. In the study of Weissmann et al. [115], a non-synonymous mutation of *PTPN11* (c.127C>T, exon 2) was identified in a subject with non-syndromic complete AVCD. Mutations in already known “syndromic” genes were also found in the study of D’Alessandro et al. [111], which enrolled a large number of subjects with complex CHD including AVCD. They found variants in six genes (*NIPBL*, *CHD7*, *CEP152*, *BMPR1a*, *ZFPM2*, *MDM4*) already known for their association with CHDs. *NIPBL*, *CHD7*, and *CEP152* are “syndromic genes” which have a consistent role in the pathogenesis of specific syndromes, namely Cornelia de Lange, CHARGE, and Seckel syndromes, respectively. However, they can show rare variants in isolated CHDs. The role of all these identified variants is still largely unknown. One important question is whether all those variants play a causal or a contributory role in the pathogenesis of non-syndromic AVCD. The possible role of large de novo genomic rearrangements such as copy number variants (CNVs) in non-syndromic AVCD was supported by the study of Priest et al. [116], which identified two rare sub-chromosomal deletions in two unrelated probands with sporadic AVCD. The 3q26 deletion contained two micro-RNA genes known as enhancers of heart transcriptions, whereas the 20p12.3 deletion contains three protein genes: *HAO1*, *TMX4*, and *PLCB1*. In particular, *PLCB1* is a regulator of cardiomyocyte hypertrophy. Thanks to the wide spread of novel techniques like exome-sequencing and array-CGH, it is possible to increase the specificity of the genotype–phenotype correlation. In the study of Priest et al. [117], they observed de novo variations in new and known genes (*NR1D2*, *ADAM17*, *RYR1*, *CHRD*, *PTPRJ*, *IFT140*, *ATE1*, *NOTCH1*, *NSD1*, *ZFPM2*, *MYH6*, *VCAN*, *SRCAP*, *KMT2D*, *NOTCH2*, *BBS2*, *EHMT1*) associated with human non-syndromic AVCD. All these analyses suggest that de novo mutations can contribute by a small fraction to the risk of isolated CHDs, including AVCD, in addition to inherited rare variants. The above-described genetic heterogeneity highlights the fact that isolated non-syndromic AVCD could result from multiple variants in different genes cosegregating together in addition to parental and environmental risk factors. Some of these genes are directly or indirectly involved in lateralization mechanisms.

## 3. Transposition of the Great Arteries

**Figure 2:**
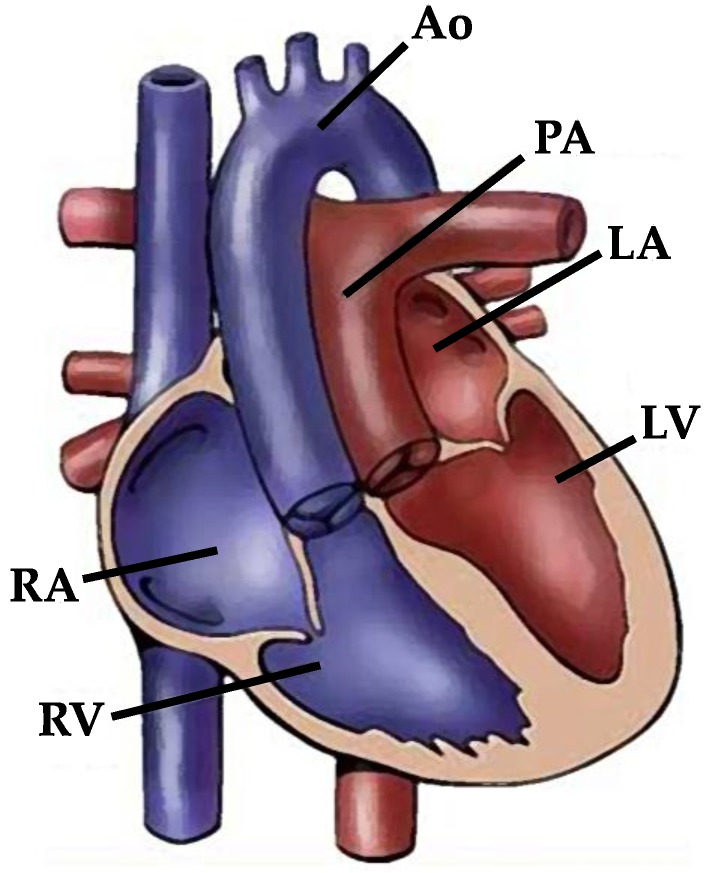
Diagram of TGA with ventriculo-arterial discordance and without spiralization of the great arteries. RA: right atrium; RV: right ventricle; LA: left atrium; LV: left ventricle; Ao: aorta; PA: pulmonary artery.

Transposition of the great arteries (TGA) accounts for 5% to 7% of all CHDs [118] and 34% of conotruncal defects with *situs solitus* [119], with a prevalence rate of 3.54 per 10,000 live births in Europe. The incidence is likely higher in the fetal population, because termination of pregnancy is not included in most population studies. TGA is the second most frequent cyanotic CHD after tetralogy of Fallot [120], and if not treated, it is the leading cause of cardiac death in neonates and infants [118,119]. TGA displays a ventriculo-arterial discordance with the aorta arising anteriorly and right-sided from the right ventricle and the pulmonary artery arising posteriorly and left-sided from the left ventricle. In contrast to the normal heart in which both outflow tracts and great vessels show a dextral (right-handed) spiralization, in TGA the great vessels present parallel course lacking normal spiralization. As a consequence of discordant ventriculo-arterial connection, the systemic and pulmonary circulations are not in series, as usual, but in parallel, with the deoxygenated systemic venous blood returning to the aorta through the right ventricle and the oxygenated pulmonary venous blood returning to the pulmonary artery via the left ventricle (Figure 2). Prenatal or neonatal diagnosis and an early arterial switch operation not only reduce mortality but also improve neurodevelopmental outcomes [118,121].

Among all CHDs, TGA may represent the most intriguing and mysterious CHD for the following reasons: its morphology is not observed either ontogenically or phylogenically; it does not represent an alternative physiological model of blood circulation; and its etiology and morphogenesis are still largely unknown [119].

### 3.1. Embryology and Pathogenetic Theories

The precise etiology of TGA is still unknown and its pathogenesis is controversial, especially because TGA is difficult to reproduce with animal models. The embryological mechanism of TGA has traditionally been explained by two main theories. Goor and Edwards formulated the first one in the 1970s: TGA can be considered as the result of an abnormal resorption or underdevelopment of the subpulmonary muscular conus with an abnormal persistence of the subaortic muscular conus. This phenomenon leads to a lack of the normal clockwise rotation of the aorta toward the left ventricle when the heart is viewed from above [122]. This “infundibular theory” can also explain the association of TGA with ventricular septal defect or a certain degree of pulmonary overriding, which are morphologically similar to double outlet right ventricle (DORV) with anterior aorta or side-by-side great arteries. Therefore, following this theory, TGA would represent the extreme form of “dextroposition of the aorta”, which goes from different forms of DORV, through tetralogy of Fallot, up to malalignment type of ventricular septal defects [123]. On the other hand, this theory is less helpful to explain the cases of TGA with intact ventricular septum. De la Cruz proposed the second theory, which is focused on the abnormal spiralization of the aorto-pulmonary septum. TGA would be the result of a linear rather than spiral development of the aorto-pulmonary septum, which puts the fourth aortic arch (the future aorta) in contact with the anterior muscular conus of the right ventricle [124,125]. However, this “extracardiac theory” does not account for the great variability of the morphology of the infundibulum, which characterizes some forms of TGA [126,127].

A recent study on chick embryos demonstrated that spiraling migration of the right SHF is required for elongation and appropriate alignment of the cardiac outflow tract. In particular, the right segment of SHF spirals posteriorly and to the left of the conotruncal junction forming the pulmonary outflow tract and most of the right ventricle in their right-handed spiral pattern [128]. Several experimental studies support the pivotal role of *Pitx2* at the arterial pole of the developing heart, which can explain the underlying mechanism of TGA, even in human beings. A *Pitx2-Wnt11* pathway regulates the outflow tract elongation by affecting extracellular matrix composition, cytoskeletal rearrangements, polarized cell movements [129], and regional proliferation [130,131]. Moreover, Bajolle et al. demonstrated that *Pitx2* drives the counterclockwise rotation of the outflow tract and that *Pitx2* mutant embryos present conotruncal defects with rotational anomalies including TGA, confirming the importance of the spiral movement of the outflow tract [132]. Because *Pitx2* is involved in left–right signaling, these experimental results suggest that embryonic laterality affects rotation of the myocardial wall during outflow tract development [132]. Additionally, the counterclockwise rotation of the outflow tract leads to local hemodynamic modifications [133], resulting in left-sided formation of the aortic arch and the regression of its right counterpart [134]. Hence, *Pitx2* (although indirectly) is able to influence the embryogenesis of the heart and great arteries by also acting on hemodynamics [35]. So, we can suggest that the definitive anatomy of the heart, characterized by the typical right-handed spiralization of the great arteries, is due to the confluence of the clockwise spiralization of the pulmonary artery with the counterclockwise spiralization of the pulmonary infundibulum. These two inverse spiralizations result in an interlock, which is favorable to correct circulation. Costell et al. reported a high incidence (11 out of 15 late embryos studied) of TGA with intact ventricular septum in transgenic mice mutated for *Perlecan*, which is a heparin-sulfate proteoglycan expressed in the basal surface of myocardium and endocardium as well as surrounding neural crest cells in wild-type embryos. In this animal model, TGA appears to be the result of an excess of mesenchyme at earlier stages of conotruncal development precluding proper outflow tract rotation and spiralization, and thus producing discordant ventriculo-arterial connection [135]. Impairment of the very complex orchestration of remodeling and alignment of the outflow tract and great arteries can result in TGA, which can represent a feature of abnormal cardiac laterality [136].

### 3.2. Genetic Syndromes and TGA

The occurrence of TGA in genetic syndromes is extremely rare. However, sporadic reports of TGA do exist in well-known genetic conditions such as monogenic disorders Noonan, Turner, Williams, and Marfan syndromes. Although TGA is considered to be virtually absent in Down syndrome [137], McCrossan and McCay recently reported a case of TGA, ventricular septal defect, and pulmonary stenosis in a patient with trisomy 21 [138] However, TGA can be sporadically associated with VACTERL association and CHARGE (coloboma, heart defects, atresia of choanae, retardation of growth, genital defect, ear anomalies) syndrome, with trisomy 8 and 18 [10,139], as well as with tuberous sclerosis [140], deletion of the long arm of chromosome 11 [141], and of the short arm of chromosome 18 [142]. According to the pathogenetic classification of CHDs proposed by Clark and more recently by Botto et al. [143], TGA is included among conotruncal heart defects, which are the result of abnormalities of ectomesenchymal tissue migration from the neural crest, often associated with 22q11DS. However, experimental studies have demonstrated that the ablation of neural crest in chick embryos very rarely results in TGA [144], and clinical studies showed that only 1% of patients with TGA have 22q11DS [145]. These observations suggest that TGA cannot be considered a typical conotruncal defect of 22q11DS, such as tetralogy of Fallot, truncus arteriosus, and interrupted aortic arch type B, arguing that the morphogenesis of this cardiac defect is probably different [146,147,148,149].

The only genetic condition with a very strong association with TGA is *heterotaxy* and in particular *right isomerism*. TGA is also quite common in cases of isolated dextrocardia with *situs solitus*, displaying a relation with defect of visceral situs [150]. In the setting of *heterotaxy*, TGA is associated with other severe cardiac malformations, and in almost 100% of cases of *right isomerism* it is associated with the complete form of AVCD [150,151] both in ventricular D-loop and in ventricular L-loop. On the contrary, TGA is rarely associated with *left isomerism*, in which the great arteries are usually normally or “inversely” related [150,152]. Even in animal models of *heterotaxy*, TGA has been commonly reported with ventricular D-loop as well as with ventricular L-loop [136,153,154]. Several families have been reported in which some members had *heterotaxy*, whereas other members exhibited isolated CHDs, including TGA [155]. Notably, in some large families with recurrence of *heterotaxy* and mutations of the gene *ZIC3* [7,156,157] (besides cases with *situs inversus*, and others with *right* or *left isomerism*), there were members with congenitally corrected transposition of the great arteries (CCTGA) with *situs solitus*. Thus, it is possible to hypothesize that in these families the same genetic mechanism could produce different phenotypes, including not only *situs inversus*, *right* or *left isomerism*, but also CCTGA with *situs solitus*.

TGA with or without *right isomerism* of the lungs has been reported in mice mutated in two of the most important genes involved in the process of laterality determination, *Smad2* and *Nodal* [158]. In some patients, a few “laterality genes” associated with *heterotaxy*—specifically *ZIC3* [157,159], *CFC1* (encoding the CRYPTIC protein) [160,161], and *Nodal* [162]—were found mutated in isolated TGA with *situs solitus*. All these clinical observations and animal models suggest that TGA might relate to laterality defects confined to the heart, in the absence of other typical features of *heterotaxy*. Mutation in the α-cardiac myosin heavy chain 6 (*MYH6*) gene was identified in a 16-year-old girl with TGA. Her mother, with a persistence of the foramen ovale, and her unaffected grandmother carried the mutation. The mutation changed a highly conserved histine to a glutamine (p.His252Gln) [163]. An interesting link between TGA and cilia is the recent study of Zahid et al. [164]. They observed a high prevalence of ciliary motion abnormalities and low nasal nitric oxide in patients with isolated TGA without *heterotaxy* or PCD and with exclusion of cystic fibrosis. These results suggest that patients with isolated TGA with ciliary dysfunction do not have PCD, but nevertheless may suffer from milder airway clearance deficiency.

### 3.3. Epigenetic Maternal Risk Factors and Experimental Animal Models

Some maternal risk factors involved in human TGA are postulated. Loffredo et al. reported an association of maternal exposure to pesticides (herbicides and rodenticides) during the first trimester of pregnancy with TGA in their infants [165]. Cases of TGA associated with intake of antiepileptic [166], hormonal [167], ibuprofen, ionizing radiation [10], and other medications [168] have been anecdotally reported. The prevalence of TGA is higher in infants of diabetic mothers [169,170] as well as in cases of in vitro fertilization [171]. On the other hand, the periconceptional use of folic acid reduces the risk of CHDs, including TGA, and its intake is recommended as a protective factor against a large spectrum of congenital malformations [172].

The most consistent method to reproduce TGA in animal models is treating pregnant mice with retinoic acid, which is the active metabolite of vitamin A, or with retinoic acid inhibitors [119,153,173]. Because the developing cardiovascular system is particularly sensitive to different levels of retinoic acid, it appears that its administration in different time points of pregnancy produces different cardiac phenotypes. In fact, mouse experiments produced not only cases of TGA with ventricular D-loop, but also some cases of TGA with ventricular L-loop (CCTGA). The administration of trans-retinoic acid to pregnant mice at E8.5 of gestation resulted in three-quarters of the fetuses presenting with TGA [174]. In another experiment, the mice embryos treated with retinoic acid at day 6.5 presented *heterotaxy* [153,173]. Therefore, it seems that there is a common pathogenetic mechanism, suggesting the presence of a relationship among these morphologically different cardiac defects.

Our group obtained TGA by the administration of BMS-189453, a retinoic acid competitive antagonist, to pregnant mice, demonstrating that critical levels of retinoic acid must be present for the normal alignment between the great arteries and the outflow tract [175]. Timing of the administration of BMS-189453 seems to be critical, since its administration before or after 7.5 days postcoitum results in much lower rates of TGA. Subsequently, we demonstrated that supplementation of folic acid and methionine to pregnant mice decreased the teratogenic effects of BMS-189453. The incidence of TGA was significantly reduced in embryos treated with folic acid compared to embryos treated with BMS-189453 [176]. In order to better identify genes/transcripts involved in the pathogenesis of CHD (including TGA) in our mouse models, we performed a global microarray analysis on embryos. *Hif1α* (hypoxia-inducible factor 1 alpha subunit) was found to be down-regulated in mice treated with BMS-189453 compared to wild-type, but up-regulated in embryos supplemented with folic acid [177]. *Hif1α* plays an essential role during heart development, and one of its downstream targets *Cited2* is involved in left–right determination. Since mutations of both of these genes cause defects in left–right patterning, and the main CHD in our mouse models was TGA, these results support the pathogenetic link between TGA and lateralization defects with *heterotaxy*.

### 3.4. Familial Recurrence in TGA

Familial cases of TGA are considered exceptional with a low risk of recurrence. The English multicentric study conducted by Burn et al. [97] reported no familial cases of TGA. In a multicentric Italian study, our group reported different epidemiologic data: the recurrence rate in siblings of patients with TGA was 1.7%. It is noteworthy that in some families, beside members with TGA, there were first-degree relatives (siblings or parents) with CCTGA. This familial clustering of TGA and CCTGA could be explained with a monogenic inheritance (autosomal dominant or recessive) with a variable phenotypic expression. In a group of familial TGA not associated with *heterotaxy*, we screened some genes that are known to be related to a subset of laterality defects and participate or cooperate in the Nodal signaling pathway, including *Zic3*, *Acvr2B*, *LeftyA*, *CFC1*, *Nodal*, *Nkx2.5*, and *Creld1*. We also screened the *GATA4*, which was previously found mutated in cases of *dextrocardia* [178], *GDF1* [179] and *FOXH1* [180], two genes linked to human CHDs, including TGA. Mutation analysis allowed the identification of three sequence variations in two out of seven TGA probands. The first patient showed double variants in two genes: namely, *FOXH1* (Pro21Ser) and *ZIC3* (Gly17Cys). The second patient showed a splice site variant (IVS2-1G→C) in *Nodal* [181]. These results provide evidence that some cases of familial TGA are due to mutations in genes related to laterality defects, confirming a pathogenetic relation between TGA and *heterotaxy*.

### 3.5. Heart and Shell: An Evolutionary Hypothesis

While the great arteries display a dextral (right-handed) spiralization in individuals in *situs solitus*, in subjects with *situs inversus*, the spiralization of the great arteries is mirror-imaged with a left-handed spiral pattern. In patients with TGA, with or without *right isomerism*, any spiral pattern of the great arteries is lost. Based on these morphological observations, we previously noted that the same right-handed spiralization is characteristic, although not exclusive, of other organisms, such as shells, some bacteria including *Bacillus subtilis*, and some climber plants including *Convolvulus arvensis*. Thus, we have suggested that the normal (right-handed) spiral pattern of the great arteries and the dominant right-handed spiral pattern of snail shells show some phenotypic similarities, arguing that human beings and shells could share a common and ancestral genetic mechanism [150]. *Nodal*, which is a transcription growth factor of the TGF-β family, plays a pivotal role in early embryonic development including mesoderm and neural induction and left–right axis in vertebrates [182,183,184,185,186]. In mouse models, it has been demonstrated that asymmetric left-sided expression of the Nodal pathway genes in lateral plate mesoderm (LPM) is essential for normal left–right body chirality. In fact, *Nodal* is constantly expressed in right LPM, the opposite side from normal, in mice with *situs inversus*. Loss of function or abnormal expression of *Nodal* generates randomization of left–right patterning of visceral organs in *heterotaxy* and TGA [186]. Mutations in the *Nodal* gene have been reported in children with *heterotaxy* [160] as well as in those with sporadic [162] and familial TGA [181]. Recent studies corroborate our observations, demonstrating the role of Nodal signaling in left–right asymmetry in snails [187,188]. *Nodal* is expressed on the right side of the embryo in the dextral (right-handed) species *Lottia gigantea*, while it is expressed on the left side in the sinistral (left-handed) species *Biomphalaria glabrata* [189]. As in vertebrates, in which the heart is the first organ to break the symmetry in a developing embryo with the onset of ventricular D-looping, in snail shells the pattern of chirality (right-handed vs. left-handed) is a sign of *situs* and of their internal organs arrangement [189,190,191]. Moreover, pharmacological inhibition of the Nodal pathway causes loss of shell coiling in snails, which results in a straight, non-spiralized shell, but, interestingly, not reverse coiling [188]. Thus, other factors upstream of *Nodal* are involved in the generation of chirality [189]. Finally, Nodal signaling appears to be a conserved pathway involved in the normal and abnormal morphogenetic mechanism of spiral coiling of the shells and the spiral pattern of the cardiac outflow tract and of the great arteries [160,186,188].

## 4. Conclusions

Chamber formation, along with in-series alignment of atria, ventricles, and outflow tracts, as well as the position of the heart relative to the midline, are all features of heart development, and are strictly dependent on patterning of the left–right axis during embryonic growth. Clinical observations made in human beings and studies in animal models of laterality disease suggest that all these stages of cardiac development are influenced by the embryonic left–right body axis. In mouse models, the disruption of pathways with a critical role for establishing left–right asymmetry often produces an abnormal rotation of the great arteries, resulting in TGA. Moreover, because isolated TGA in *situs solitus* has been found in transgenic mice as well as in human beings harboring mutations in laterality genes, this suggests that TGA may be the only phenotype of a left–right patterning defect.

Cilia play an essential role in generating left–right asymmetry, and recent studies show that cilia and morphogenesis of the heart are very intimately associated. Indeed, cilia are largely expressed in the embryonic heart, including in the atrial and ventricular myocardium, the atrioventricular and outflow endocardial cushions. This is further supported by the recovery of pathogenic mutations in genes involved in cilia-transduced cell signaling, including mutations in genes involved in the *Shh* signaling pathway, which plays an important role in cardiovascular development. *Shh* signaling is active at the venous pole of the SHF, and it is essential for normal development of DMP and atrioventricular canal formation and septation. The link between cilia, the *Shh* pathway, and AVCD is particularly evident in patients with Ellis–van Creveld syndrome or with polydactyly syndromes, who display, in *situs solitus*, AVCD associated with other CHDs that are strongly reminiscent of the cardiac phenotype found in *heterotaxy*, especially in *left isomerism*.

In conclusion, AVCD and TGA are to be considered as complex and heterogeneous CHDs from clinical, prognostic, phenotypical, and molecular points of view. Thanks to the novel approaches applied to cardiogenesis, an interesting step into the knowledge of the molecular pathogenesis has been made. This has led to the suggestion that some isolated heart malformations, even in the setting of *situs solitus,* may actually be included in the spectrum of laterality defects. Genetics and pathogenetic mechanisms involved in laterality development deeply influence the first steps of cardiac embryology. Therefore, we can hypothesize that other types of CHDs—in particular those with ventricular L-loop and those with major distortion of ventricles and great arteries—can result in relation to lateralization defects.

Finally, we believe that the amazing and exciting advances made in the fields of heart embryology and molecular genetics in recent years, besides bettering our understanding of the mechanisms underlying CHDs, also give us the opportunity to modify their classification, which is still based mostly on anatomic and hemodynamic characteristics. As a matter of fact, in our opinion it is better to have an in-depth understanding of the main abnormal events of cardiac morphogenesis that lead to cardiovascular malformations, rather than merely rigidly classifying them.

## Figures and Tables

**Table 1:** Cardiovascular malformations in heterotaxy.

Right Isomerism	Left Isomerism
Levocardia/Mesocardia/Dextrocardia	Levocardia/Mesocardia/Dextrocardia
Inferior vena cava and aorta are ipsilateral and lie together on the right or the left side of the spine	Interrupted hepatic portion of the inferior vena cava with azygos continuation
Persistent left superior vena cava	Persistent left superior vena cava sometimes with unroofed coronary sinus
Absence of coronary sinus	
Total anomalous pulmonary venous return (usually extracardiac)	Partial anomalous pulmonary venous return
Common atrium with virtually absent atrial septum and with right bilateral morphology of the atrial appendages	Common atrium or ostium primum atrial septal defect with left bilateral morphology of the atrial appendages
Complete atrioventricular canal defect	Partial atrioventricular canal defect
Ventricular D- or L-loop	Ventricular D- or L-loop
Dominant ventricle (usually of right ventricular type) with malalignment of the atrioventricular canal	Balanced ventricles
	Rarely dominant ventricle
Double-outlet right ventricle (or transposed great arteries) with an anterior aorta and parallel great arteries associated with pulmonary stenosis or atresia and hypoplasia of the infundibular septum	Normally related (or inverted normally related) great arteries
Pulmonary stenosis or atresia (>90% of cases)	Pulmonary stenosis or atresia (~30% of cases)
Right-sided aortic arch	Left-sided obstructions including mitral valve stenosis and aortic coarctation (~25% of cases)
Bilateral sinus node	Hypoplastic or absent sinus node
	Single/paired atrioventricular nodes
Paired (anterior/posterior) atrioventricular nodes with sling formation	Interruption between atrioventricular node and His bundles

**Table 2:** Genes involved in the development of atrioventricular canal defect (AVCD) and transposition of the great arteries (TGA).

Gene	Chromosome	Proposed impact
*DNAH5*	5p15.2	Cilium movement, determination of left/right asymmetry
*DNAH11*	7p15.3	Cilium movement, determination of left/right asymmetry
*DNAI1*	9p13.3	Cilium movement, determination of left/right asymmetry
*IFT25*	1p32.3	Intraciliary transport, dynamic transport of Shh signaling molecules within the cilium
*IFT88*	13q12.11	Cilium assembly
*IFT172*	2p23.3	Intraciliary transport involved in cilium assembly
*FUZ*	19q13.33	Cilium assembly, regulation of smoothened signaling pathway
*KIF7*	15q26.1	Ciliary basal body, regulation of smoothened signaling pathway
*CP110*	16p12.3	Ciliary basal body organization, regulation of cilium assembly
*MKS*	17q22	Cilium assembly, regulation of smoothened signaling pathway involved in dorsal/ventral neural tube patterning
*FOXF1*	16q24.1	Endocardial cushion development, regulation of smoothened signaling pathway
*HOXD13*	2q31.1	Skeletal system development, Shh signaling
*CRELD1*	3p25.3	Cardiac septum development, endocardial cushion development
*NR2F2*	15q26.2	Forebrain, limb development, endocardial cushion development
*GATA4*	8p23.1	Cardiac morphogenesis, second heart field (SHF) contribution
*PTPN11*	12q24.13	Heart development
*NIPBL*	5p13.2	Heart morphogenesis
*CHD7*	8q12.2	Atrioventricular canal development
*CEP152*	15q21.1	De novo centriole assembly involved in multi-ciliated epithelial cell differentiation
*BMPR1a*	10q23.2	BMP signaling pathway involved in heart development
*ZFPM2*	8q23.1	Ventricular septum morphogenesis
*MDM4*	1q32.1	Endocardial epithelial-to-mesenchymal transition regulation
*PLCB1*	20p12.3	Positive regulation of embryonic development
*PITX2*	4q25	Determination of left/right asymmetry, atrioventricular valve development
*ZIC3*	Xq26.3	Determination of left/right asymmetry
*CFC1*	2q21.1	Determination of left/right asymmetry
*MYH6*	14q11.2	Ventricular cardiac muscle tissue morphogenesis
*NODAL*	10q22.1	Determination of left/right asymmetry
*HIF1A*	14q23.2	Heart looping
*CITED2*	6q24.1	Heart development, determination of left/right asymmetry
*NKX2.5*	5q35.1	Heart looping, heart morphogenesis
